# Assessing young Kenyan women's willingness to engage in a peer-delivered HIV self-testing and referral model for PrEP initiation: A qualitative formative research study

**DOI:** 10.3389/fpubh.2022.932948

**Published:** 2022-10-06

**Authors:** Maureen McGowan, Edinah Casmir, Njeri Wairimu, Peter Mogere, Albrecht Jahn, Kenneth Ngure, Katrina F. Ortblad, Stephanie D. Roche

**Affiliations:** ^1^Heidelberg Institute of Global Health, Heidelberg University, Heidelberg, Germany; ^2^Kenya Medical Research Institute, Nairobi, Kenya; ^3^Partners in Health and Research Development, Thika, Kenya; ^4^School of Public Health, Jomo Kenyatta University of Agriculture and Technology, Nairobi, Kenya; ^5^Public Health Science Division, Fred Hutchinson Cancer Research Center, Seattle, WA, United States

**Keywords:** HIVST, PrEP, peer delivery, AGYW, sub-Saharan Africa

## Abstract

**Background:**

Pre-exposure prophylaxis (PrEP) is highly effective for HIV prevention, but uptake remains low, especially among adolescent girls and young women (AGYW) in Kenya. A model in which trained AGYW using PrEP deliver HIV self-tests to their close friends and refer them to PrEP may help increase PrEP uptake in this population. To understand AGYW's potential willingness to engage in such a model, we conducted a qualitative formative study in Kenya.

**Method:**

We conducted semi-structured in-depth interviews (IDIs) with AGYW (16 to 24 years) in Kiambu County who were determined at risk of HIV acquisition. We purposively recruited “PrEP-naïve” (no prior PrEP use) and “PrEP-experienced” AGYW who used PrEP for at least 1 month within the previous year. We solicited perspectives on initiating/engaging in conversations about HIV risk and PrEP, distributing/receiving HIV self-test kits, and referring/following through on a referral to clinic-based HIV services. We analyzed verbatim transcripts using rapid qualitative analysis and a combination of inductive and deductive approaches, with the latter informed by the Integrated Behavior Model (IBM).

**Results:**

From August to December 2020, we conducted 30 IDIs: 15 with PrEP-experienced and 15 with PrEP-naïve AGYW. Participants' median age was 20 [interquartile range (IQR): 20–22]. Overall, most participants anticipated that they would be willing to engage in this model. PrEP-experienced AGYW emphasized the salience of their concerns about friends' HIV risk behaviors, with several noting that they are already in the habit of discussing PrEP with friends. Many additionally expressed positive attitudes toward the proposed target behaviors, perceived these to be normative among AGYW, and expressed confidence in their ability to carry out the behaviors with proper support. Although few participants had HIVST experience, nearly all anticipated they would be able to use an HIV self-test kit correctly if provided instruction.

**Conclusion:**

The Kenyan AGYW who participated in this study generally anticipated that they would be willing to engage in a formal peer PrEP referral model enhanced with peer-delivered HIV self-tests. Future research is needed to pilot test this model to determine its acceptability, feasibility, and effect on HIVST and PrEP uptake within this population.

## Introduction

Although Kenya has made immense progress in the fight against HIV in recent decades, progress has been uneven, and adolescent girls and young women (AGYW) remain at disproportionally high risk of HIV infection (1, 2). Between 2018 and 2019, Kenyan AGYW ages 15 to 24 years accounted for twice as many new HIV infections as their male counterparts (2, 3), and in 2020, were estimated to contribute to roughly a third of the country's new ~40,000 HIV infections (1). Drivers of HIV risk among AGYW in Kenya include engagement in sexual behaviors associated with HIV risk acquisition, such as intergenerational and transactional sex; low perception of HIV risk; high rates of gender-based violence; early discontinuation of school; alcohol and drug use; and structural barriers to HIV prevention services, such as economic dependency on male partners and limited access to healthcare services (1, 3, 4). Additionally, limited routine HIV testing hinders young women from knowing their HIV serostatus and being linked to appropriate HIV prevention or treatment services (5–7).

One approach demonstrated to enhance uptake of routine HIV testing among young women in East Africa has been HIV self-testing (HIVST) (8, 9). Several studies from sub-Saharan African (SSA) countries that offered HIVST to populations with high HIV risk—including AGYW (10–12), female sex workers (FSWs) (13, 14), and men who have sex with men (MSM) (15, 16)—found HIVST to be acceptable and appropriate. Participants in these studies often reported liking HIVST and, in some cases, preferring it over traditional facility-based HIV testing because of greater privacy and confidentiality of results; convenience and lower opportunity costs; greater chance to test with partners; and increased sense of empowerment and control over one's health (8–10, 12, 15–17). Two recent studies conducted among Kenyan AGYW found that most participants thought HIVST kits were easy to use and were able to perform HIVST correctly and with confidence (10, 12).

Other studies have found that uptake of HIV prevention services is enhanced when delivered through peer networks (18, 19). Hypotheses for this include that peers are trusted members of the target community; able to identify intervention candidates; utilize appropriate language to communicate sensitive topics; provide social support; and are capable of mitigating structural barriers to facility-based healthcare (e.g., transportation) (8, 18–20). Several studies conducted among key populations, including MSM (21, 22) and FSW (13, 14), have successfully implemented peer referral and peer-delivered HIV prevention interventions. Preliminary evidence suggests that sub-Saharan African AGYW may be open to delivering HIVST to peers and assisting them with use (8, 23) and may also encourage their peers to uptake PrEP (24, 25); however, few studies have investigated whether combining peer-delivered HIVST and peer referral to PrEP might increase PrEP uptake among adolescent girls and young women.

To leverage and formalize existing peer referral practices, we will conduct a pilot study to test the effect of a peer PrEP referral model enhanced with HIV-self test delivery on HIV testing and PrEP uptake among Kenyan AGYW. In this model, PrEP-experienced AGYW will be trained to distribute HIVST kits to their PrEP-naïve peers and encourage them to seek PrEP at a clinic if they test negative or HIV treatment if they test positive. To inform the design of this model, we conducted formative qualitative research to understand PrEP-experienced and PrEP-naïve Kenyan AGYW's intention to engage in this intervention as peer providers or clients, respectively, and to identify factors that may help or hinder their decision to engage. Findings from this formative research will be incorporated into the model to be tested in the pilot study, with the intent of increasing the intervention's appropriateness and acceptability.

## Methods

### Study setting

This study was conducted in Kiambu County, a peri-urban area in central Kenya, which includes major industrial hubs in Thika, Ruiru, Limuru, and Kikuyu, as well as low-resourced areas including the Kiandutu Slums (26–28). Kiambu County has been designated by the Kenya Ministry of Health as a priority area for HIV prevention (1). Kiambu County is home to approximately a quarter of a million AGYW ages 15 to 24 (29, 30), and this population accounts for over 1,000 new HIV infections annually (1, 31).

### Study design and sampling

We conducted 30 semi-structured in-depth interviews (IDIs) with AGYW ages 16 to 24 years who self-reported being HIV-negative, were at high risk of HIV infection as per the Kenya Rapid Assessment Screening Tool (RAST) and either had never before used PrEP (hereafter, “PrEP-naïve”) or had used PrEP for at least 1 month within the last year and self-reported having had good adherence to PrEP (hereafter, “PrEP-experienced”). Study participants were recruited through purposive sampling. To recruit PrEP-experienced and PrEP-naïve AGYW, community health volunteers affiliated with Kiambu County public healthcare facilities and youth-friendly HIV prevention programs approached prospective participants, provided them with an overview of the study, and, if interested in participating, referred them to a Kenyan qualitative researcher (author EC) from Partners in Health & Research Development (PHRD), a Thika-based research organization affiliated with the Kenya Medical Research Institute. Prospective participants were also recruited in part from previous research studies at the PHRD or referred by other prospective participants. EC then screened participants for eligibility and administered informed consent.

This study was approved by the Institutional Review Board of the University of Washington (STUDY00009127) and the Scientific Ethics Review Unit (SERU) of KEMRI (0164/4004). All participants provided written informed consent. Participants were compensated 500 Kenyan shillings [~5 United States Dollars (USD)] for interview completion.

### Data collection

Authors MM, EC, NW, KN, KFO—all of whom have graduate-level training in qualitative research and two of whom have over a decade of experience conducting PrEP research in the study setting—developed *de novo* semi-structed IDI guides—one for PrEP-naïve and one for PrEP-experienced participants. These guides were informed by the Social Ecological Model, a framework that considers the interplay among individual, relationship, community, and societal factors that influence health behavior (32, 33).

Each guide was pilot tested with young female PHRD staff and revised for clarity. The final guides solicited participants' perspectives on the respective target behaviors of peer providers and clients and factors that might influence their use of target behaviors, such as their knowledge of and perceptions about PrEP and HIVST (Appendix 1). All interviews were conducted by author EC in the participant's preferred language (English or Kiswahili) in a private room at PHRD's office. Interviews were audio recorded with participant consent and transcribed verbatim, with Kiswahili content simultaneously translated to English, as needed.

### Data preparation and analysis

We analyzed the data using rapid qualitative analysis principles (34, 35) that employed a combination of inductive and deductive approaches, the latter informed by the Integrated Behavior Model (IBM) (36) a conceptual model for understanding determinants of behavioral intention. Drawing on the Theory of Reasoned Action and the Theory of Planned Behavior (36), the IBM posits that individuals' intention to perform a behavior or set of behaviors (hereafter, “target behaviors”) is determined by their attitudes, normative beliefs, and sense of personal agency. According to the IBM, attitudes are influenced by one's feelings and beliefs about the target behaviors; normative beliefs are shaped by one's perceptions about *other people*'*s* beliefs and expectations related to the target behaviors; and sense of personal agency is determined by one's beliefs about how much control and ability they have to perform the target behaviors.

**Figure 1:**
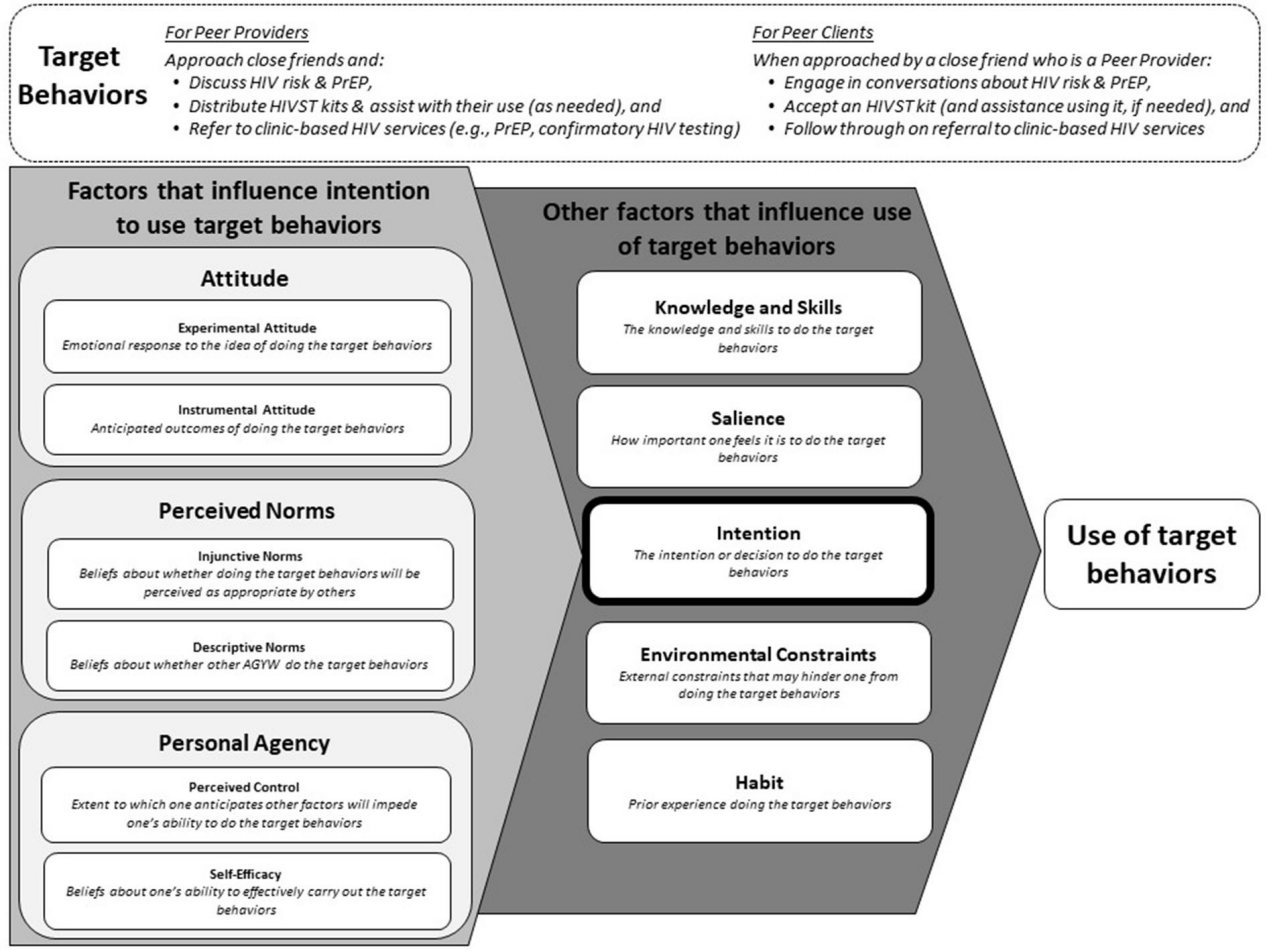
Modified Integrated Behavior Model showing factors hypothesized to influence whether PrEP-experienced and PrEP-naïve AGYW perform the target behaviors of a peer provider or peer client, respectively, within the proposed model.

Our analytic framework, modified for target behaviors of interest, is depicted in Figure 1. When analyzing the transcripts of PrEP-experienced participants, the target behaviors were framed as broaching the topics of HIV risk and PrEP with a peer; offering this peer a free HIVST kit, instructions on how to use the kit, and (if desired) assistance conducting the HIVST; and encouraging this peer to seek PrEP at a clinic if she tested negative or to seek confirmatory HIV testing and, if necessary, HIV treatment if she tested positive. When analyzing the transcripts of PrEP-naïve participants, the target behaviors were framed as agreeing to engage in discussions initiated by a peer about one's HIV risk and PrEP; accepting an HIVST kit from this peer; and following through on the peer's advice to seek additional services (e.g., PrEP, confirmatory HIV testing) at a clinic.

After repeated readings of the transcripts, authors MM and SDR created a memo template that included sections for each component of IBM, adapted for the intervention of interest. Authors MM and SDR then each drafted analytic memos for the same three interviews. Each memo summarized the key points from the interview, noted similarities and differences from other interviews, and included illustrative quotes. Thereafter, they compared the content of these three memos and revised the memo template, as needed. All remaining memos were written by author MM and checked by SDR. Once memos were complete, MM and SDR reread all memos and summarized findings according to IBM components.

## Results

### Participant demographics

Between August and December 2020, we conducted 15 interviews with PrEP-experienced AGYW and 15 with PrEP-naïve AGYW (Table 1). Among participants, median age was 20 years [interquartile range (IQR): 20–22], and most (27/30; 90%) had a secondary level of education. Most (25/30) had a monthly household income of < 20,000 Kenya Shillings [~$185 US Dollars (USD)], and about half (16/30) had one or more children. Most participants (26/30) reported testing for HIV within the last 6 months—eight on the day of their interview.

**Table 1:** Demographic characteristics of participants.

**Characteristic**	**All**	**PrEP-experienced**	**PrEP-naïve**
	**(*n* = 30)**	**(*n* = 15)**	**(*n* = 15)**
Age (median, IQR)	20 (20–22)	21 (21–23)	20 (19–20)
Years of education completed (median, IQR)	12 (10–13)	12 (10–14)	12 (11–12)
Monthly household income, KES^a^ (*n*, %) * < 5,000* *5,000–10,000* *11,000–20,000* *21,000–30,000* *>30,000*	3 (10) 15 (50) 7 (24) 4 (13) 1 (3)	0 (0) 6 (40) 6 (40) 2 (13) 1 (7)	3 (20) 9 (60) 1 (7) 2 (13) 0 (0)
Time since last HIV test (*n*, %) * < 3 months* *3–6 months* *7–12 months* *>12 months* *Never tested*	24 (80) 2 (7) 2 (7) 1 (3) 1 (3)	12 (80) 2 (13) 0 (0) 1 (7) 0 (0)	12 (80) 0 (0) 2 (13) 0 (0) 1 (7)
Engaged in condomless sex with partner (s) of unknown or positive HIV status, past 6 months (*n*, %)	19 (63)	11 (73)	8 (53)
Exchanged sex for money/gifts, past 6 months (*n*, %)	9 (30)	6 (40)	3 (20)

Interquartile range (IQR); pre-exposure prophylaxis (PrEP); Kenyan shillings (KES).

^a^5000 KES is comparable to $46 United States Dollars, using the exchange rate of 107.76 KES = 1 USD on 1 August 2020 (exchangerates.org).

Most PrEP-experienced participants (11/15) reported being introduced to PrEP by a female friend or family member close in age to them. Duration of PrEP use among these participants ranged from 0.5 to 3 years. At the time of interview, about two-thirds (11/15) were still using PrEP, and the remaining four reported that they stopped using PrEP within the past year. Of the PrEP-naïve participants, one-third (5/15) had previously been referred to PrEP by a peer but not followed through on the referral.

Below, we summarize what PrEP-experienced AGYW and PrEP-naïve AGYW reported about the target behaviors that peer providers and clients would have to carry out in this model according to the following IBM components hypothesized to influence one's intent to perform a behavior: salience of target behaviors and attitudes toward them, perceived norms and existing habits, and personal agency and capability.

### Salience of and attitudes toward target behaviors

Many PrEP-experienced participants reported that their friends commonly engaged in risky behaviors, such as having unprotected sex with people whose HIV status they did not know. These participants described worrying that their friends might acquire HIV and that finding a way intervene was at the top of their minds (**salience**):

*Almost all [of my friends have HIV risk] because they party; they have [multiple] boyfriends; … some of them are drug users. They don't care [about their HIV risk]*. (PrEP-experienced participant #13, age 23).

*[My friends] are ignorant [about HIV]. They aren*'*t educated. … I would really love to [encourage them to test for HIV] because I feel like they are going astray. … [One friend] was like, “It's not serious.” [I said,] “It is serious because you*'*re going to have sex with him without protection, and you don*'*t know anything [about his HIV status].” … Another one had an abortion and engaged in sex 3 months after and took P2 [emergency contraception]. … [I said to her,] “So the fact that you took P2 [means] you didn*'*t have protected sex.”* (PrEP-experienced participant #2, age 23).

PrEP-naïve AGYW similarly reported that their friends were at risk of HIV acquisition, with some expressing fear of acquiring HIV themselves:

*You might find yourself exposed to HIV either deliberately or not deliberately, … like you can engage in sex that you had not planned, so everybody is at risk [of HIV]*. (PrEP-naïve participant #2, age 23).

Perhaps in light of these concerns, most PrEP-experienced participants expressed a positive emotional response (**experiential attitude**) to the prospect of approaching their close friends to discuss HIV, offering them HIVST kits, and connecting them to HIV prevention or treatment services, if needed. Referencing their feelings of care for their friends, nearly all participants viewed the behaviors of a peer provider as a potential way to positively influence their friends' lives:

*I would [like talking to my friends about HIV] because I care about them. I would not want to hear that they get infected [with HIV] when there is a prevention measure they can use to avoid being infected*. (PrEP-experienced participant #6, age 22).

*I would be comfortable [talking to my friends about HIV] because we stay together and love each other. … I would tell them it [PrEP] is a good drug which one can use to protect themselves*. (PrEP-experienced participant #3, age 21).

When asked how they would feel if a friend talked to them about HIV and PrEP and offered them an HIVST kit, most PrEP-naïve participants said they would feel cared for by their friend who, they believed, would have their best interests in mind:

*I would be happy [if my friend approached me about PrEP] since that friend is concerned about my life*. (PrEP-naive participant #1, age 20).

However, a few PrEP-naïve participants said they would view such an action from a friend with suspicion or mistrust, wondering whether their friend was implying that they already have HIV or passing judgement on their sexual behaviors:

*I would not trust her [my friend] because I wouldn*'*t know why she is asking me about it [PrEP]… [I might wonder,] “Does she think that I have HIV?”* (PrEP-naïve participant #5, age 19).

*[I would feel] nervous. Why would a friend think that I need to use [PrEP]?* (PrEP-naïve participant #15, age 19).

With respect to participants' **instrumental attitudes**—their beliefs about what would happen if they carried out the behaviors of a peer provider—most felt as though they could successfully convince some of their friends to test themselves and consider starting PrEP if they tested HIV-negative. However, noting that HIV and PrEP stigma and misconceptions are still prevalent in their communities, a few participants worried that talking to their friends about HIV and PrEP might damage their friendships and/or jeopardize their own reputations, particularly if it entailed disclosing their own PrEP use:

*There are some friends I won*'*t be comfortable telling them about PrEP. … I think it*'*s [because of] the doubts and the stigma. … If I tell them, “I*'*m doing this [taking PrEP],” they are like they do not trust you. [They ask,] “Are you sure you aren*'*t sick [HIV-positive]?” Those kinds of questions*. (PrEP-experienced participant #2, age 23).

To mitigate the risk of these negative outcomes, these participants anticipated that they would be selective and only approach their closest friends with whom they have substantial trust. By contrast, some participants were unconcerned about such outcomes, explaining that they did not fear stigmatization and/or had already disclosed their PrEP use to most of their friends:

*I don*'*t fear [disclosing my PrEP use to others] because I am protecting myself. I am not doing that [i.e., taking PrEP] for anything but for myself*. (PrEP-experienced participant #12, age 21).

*Most of them [my friends] know that I take the medicine [PrEP], so it won*'*t be hard for me to talk [to them] about it*. (PrEP- experienced participant #10, age 20).

PrEP-naïve participants generally reported being open to the idea of listening to their friends talk about HIVST and PrEP. A few participants said they might be hesitant to accept the intervention due to concerns that they would have to disclose their HIVST result and/or PrEP use to their friend (the peer provider) who, they worried, might not keep this information confidential:

*I might want to say yes [to PrEP], but you know, some friends might not be good because they will tell you that and they go telling other people that you are using PrEP*. (PrEP-naïve participant #4, age 18).

Some PrEP-naïve participants, however, said they would be more motivated to take PrEP if their friend first disclosed their own PrEP use to them and were able to reassure them that PrEP works:

*I might come for it [PrEP] if she [my friend] tells me that she uses [it]*. (PrEP-naïve participant #7, age 20).

*I would like to know what PrEP is, how long I need to use it, and if she [my friend] is 100% sure that I will not get HIV [if I use it]*. (PrEP-naïve participant #3, age 24).

Some PrEP-naïve participants reported that they would welcome being engaged in such a conversation by a friend, as this would give them an opportunity to ask outstanding questions, they had about how PrEP is used, its side effects, and where they could access it.

### Norms and habits around target behaviors

Perhaps influenced by their own experiences of learning about HIV and PrEP from friends (**descriptive norm**), most PrEP-experienced participants indicated that if they approached their close friends to talk about HIVST and PrEP, this behavior would not be perceived as inappropriate or non-normative (**injunctive norm**) unless they approached someone they did not know well or at all:

*You can*'*t begin sharing with a stranger on PrEP since you don*'*t know them*. (PrEP-experienced participant #1, age 21).

*It*'*s somehow weird to talk to them [i.e., to mere acquaintances] since … we are not that close*. (PrEP-experienced participant #15, age 23).

In fact, nearly all PrEP-experienced participants reported at least some experience (**habit**) performing the peer provider behaviors of broaching the topics of HIV and PrEP with friends and providing them with information:

*Personally, I talk to people a lot [about PrEP]. … When I*'*m together with my friends, we talk about things which are beneficial to us. … [And I say,] “Have you ever heard about a drug called PrEP?” [and I*'*ll describe] how it assists [against HIV]*. (PrEP-experienced participant #5, age 21).

When asked about how they would feel if a friend broached the topic of HIV prevention with them, no PrEP-naïve participant indicated that such behavior would be inappropriate. On the contrary, most reported that they routinely talk with their friends about sex and HIV prevention:

*[It would be easy to talk about PrEP with my friends] because I do not fear them [my friends]. Secondly, everybody knows about sex, and it is something you cannot do without. … It is not something that is being introduced*. (PrEP-naïve participant #2, age 23).

Additionally, one-third of PrEP-naïve participants (5/15) reported that their friends had previously encouraged them to initiate PrEP. A few PrEP-naïve participants even described previously telling their friends about HIVST kits and/or encouraging them to seek out PrEP, despite not using PrEP themselves:

*She [my friend] told me that she fears [HIV]. I told her that since I had a [HIV self-test] kit, I give [it to] her. She told me, “If you have it, why don*'*t you test me?” I asked her, “Are you okay with that?”… and I tested her*. (PrEP-naïve participant #2, age 23).

### Personal agency and capability to carry out target behaviors

Nearly all PrEP-experienced participants strongly believed that they would be effective at engaging their close friends in conversations about HIV and PrEP (**personal agency**). Most imagined that it would be easy to broach these topics with friends during their usual conversations about relationships, sexual behaviors, and reproductive health:

*I*'*ll ask one [of my friends], “How many boyfriends are you having?” “I*'*ll ask her if she is aware of their [HIV] status. If she says no, I*'*ll ask if she knows about PrEP. From that point, I*'*ll be able to share with her the benefits of that drug.”* (PrEP-experienced participant #7, age 20).

When asked what questions, if any, they still had about PrEP, a few participants indicated wanting to know more about side effects in the short and long term, such as whether PrEP causes weight gain or can lead to kidney damage. One participant wondered whether she was still protected against HIV despite having stopped taking PrEP 1 year earlier.

PrEP-naïve participants likewise reported having several unanswered questions about PrEP's efficacy, side effects, how and when it is used (e.g., every day or just as needed), and whether it is safe to use during pregnancy. A few participants also confused PrEP with post-exposure prophylaxis (PEP) or antiretroviral therapy (ART) and expressed concerns about long-term PrEP use causing infertility and/or making ART drugs less effective if they were to subsequently contract HIV.

With respect to HIV testing, only about a quarter (4/15) of PrEP-experienced participants had any prior experience conducting an HIVST on themselves; nevertheless, nearly all expressed high confidence in their ability to instruct and, if necessary, assist their friends with conducting and interpreting the HIVST, so long as they received training:

*I*'*ve never tested myself [using an HIV self-test], but I*'*ve seen how they go about it. It*'*s not hard. … [What would make it easier for me to do it is] just having a guide and being shown on how to do it*. (PrEP-experienced participant #3, age 21).

Similarly, few (2/15) PrEP-naïve participants had prior experience using HIVST kits; however, most anticipated they would be able to use the HIVST kits correctly if provided instruction, and nearly all liked the idea of being able to test themselves in private:

*[HIV self-testing is] better since most people have that fear that, “If I go for testing [at a clinic], doctors will know that I am [HIV-]positive.” So, it is better if one tests themselves and seeks help from healthcare professionals after [getting] the [HIVST] results*. (PrEP-naïve participant #1, age 20).

### Environmental constraints to carrying out target behaviors

PrEP-experienced participants identified two potential **environmental constraints** that could hinder them from carrying out peer provider behaviors. The first, mentioned by just one participant, was financial resources for connecting with friends:

*It might be challenging [for me to check in on my friends] because sometimes you many find that you do not have airtime, or you find that she is not on[line]*. (PrEP-experienced participant #8, age 22).

The second potential constraint, mentioned by two participants, was saturation of PrEP knowledge and/or use within their friend networks:

*[My friends] are the ones who advised me [to go] on PrEP. So, I cannot share with them [i.e., introduce them to PrEP] since they already know [about it]*. (PrEP-experienced participant #1, age 21).

*The friends I attend classes with, most of them already know about its [PrEP*'*s] benefits*. (PrEP-experienced participant #15, age 23).

PrEP-naïve participants, for their part, identified three potential constraints to them engaging in conversations about HIVST and PrEP with a friend, receiving an HIVST kit from them, and/or seeking HIV prevention or treatment services at a clinic as recommended: (1) lack of means (e.g., a phone, airtime) to have such conversations if not held face-to-face; (2) competing priorities that limit the amount of time they can spend with friends; and (3) challenges affording clinic-based care that meets their needs for convenience and privacy:

*[I have concerns about the] availability of PrEP because it is not available over the counter and most people fear going to the hospital. … [I would want to access PrEP] from a place where there is either one or two people whom I trust. You know, like in a public hospital, doctors are in shifts, so I do not trust anybody. … And there*'*s this notion in the society, if you are seen coming from CCC [the HIV clinic], then you are definitely sick [HIV-positive]*. (PrEP-naïve participant #2, age 23).

## Discussion

In this qualitative formative research study, PrEP-experienced and PrEP-naïve AGYW living in central Kenya identified several factors that may support AGYW engagement as peer providers or clients in a peer PrEP referral model with HIVST kit delivery, including: (1) positive attitudes toward the target behaviors of peer providers and clients; (2) a widespread perception that these target behaviors are normative among AGYW; and (3) generally high confidence in AGYW's ability to carry out the target behaviors with proper support. Additional factors that suggest that some PrEP-experienced AGYW would be willing to engage in this model as peer providers are the importance they place on their friends' well-being and their preexisting tendency to discuss HIV risk and PrEP in casual conversations with friends. Our study adds further evidence to the literature that informal referral to PrEP is common among AGYW and highlights how modifying existing peer referral models to zero in on smaller friend networks and enhance referrals with peer-delivered HIVST kits may be an effective strategy for reaching certain AGYW. Our findings have implications for whom to recruit as peer providers and additional implementation strategies that may be needed to further support referral follow-through.

A major barrier to PrEP uptake among AGYW in SSA is low perception of HIV risk (37). Accurate HIV risk perception is contingent, in part, on honest acknowledgment of recent sexual activity (e.g., number of sex partners, use/non-use of protection, and knowledge/lack thereof of sex partners' HIV serostatus). To help adolescents and young adults feel comfortable divulging such sensitive information, the Kenya Ministry of Health (38) and the World Health Organization (39), among others (40), have pushed for youth-friendly sexual and reproductive health services, the provision of which hinges on strong rapport-building between healthcare personnel (including peer providers) and youth. Yet, one group of people who already boast strong rapport with adolescents and frequently have access to current, accurate information about adolescents' sexual behaviors is their close friends (41). Indeed, the PrEP-experienced AGYW in our study described having a direct window into the sex lives of their close friends who often freely share details about who they are engaging in sex with and under what circumstances.

This finding gives reason for optimism that this peer-delivered intervention might be able to reach and engage AGYW at high risk of HIV by leveraging the existing rapport between peer providers and their close friends. Armed with “insider information” about their friends' recent sexual activity, some PrEP-experienced AGYW may be particularly well-positioned to identify which of their close friends have HIV risk, helping them become aware of their need for HIV prevention services, and referring them to PrEP services. By having peer providers target just their closest friends, this model could complement existing AGYW PrEP demand creation strategies, such as PrEP Champions or HIV Prevention Ambassadors, in which charismatic AGYW are recruited and trained to approach unknown peers in their community (42, 43) and refer them to PrEP services (24). As several PrEP-naïve AGYW in our study pointed out, having discussions about HIV risk behaviors within the safety of a trusting friendship may better suit AGYW who are hesitant to share details of their personal sex lives with, and/or to accept advice from, AGYW they do not know well or at all. This finding aligns with qualitative findings from a recently completed trial of peer-delivered HIVST kits in rural South Africa, in which some participants randomized to a peer navigator arm reported avoiding sharing personal information with peers due to mistrust (44).

As indicated by our study participants, the desire to protect friends from HIV will likely be a critical driver of peer providers' decision to engage in this intervention, especially if participation is unpaid. As such, implementers of this model—may wish to seek out AGYW who are: (1) deeply concerned that their friends are at imminent risk of acquiring HIV, and (2) have high intrinsic motivation to intervene, as this may help them follow through with the target behaviors even when doing so poses potential social risks for them (e.g., damage to their friendships and/or reputation). This finding resonates with literature on other lay provider models (45), which has found that a sense of purpose is critical to motivating uptake and sustained use of target behaviors.

Model implementers should also consider recruiting AGYW who are willing to disclose their own PrEP use to their close friends (the target intervention clients). Most of the PrEP-naïve AGYW in our study indicated that they would be more comfortable engaging in conversations about HIV and PrEP initiated by a friend if they knew that this individual was themselves using PrEP. Yet, in line with other studies of AGYW PrEP-users in SSA (46–49), many of the PrEP-experienced AGYW in our study described using only select disclosure (divulging their PrEP use to just a few highly trusted individuals), and some expressed hesitancy at the prospect of widening that circle. Limiting peer providers in this model to individuals who are willing to disclose their PrEP use may help mitigate challenges of credibility (distrust of health information received from non-healthcare professionals) (44) by assuring prospective intervention clients that peer providers have first-hand experience using the PrEP. It may also encourage prospective intervention clients to ask questions about PrEP, thus creating an opportunity for peer providers to dispel common misconceptions about PrEP that might otherwise keep AGYW from seeking it.

This study has limitations. First, because this was formative research, participants' perspectives were not anchored to first-hand experience with the proposed intervention and may not accurately reflect how they would feel in a real-world situation. Second, because PrEP delivery to AGYW in Kenya has, to date, largely taken place within the context of PrEP demonstration projects, we recruited some of our sample of PrEP-experienced AGYW from participants of prior PrEP research studies. However, AGYW willing to receive PrEP via a research study may differ from other PrEP-using AGYW in ways that could affect their perspectives on and willingness to engage in the model proposed in this study. Third, our analytic framework—the IBM—assumes that individual intention to perform a behavior is formed with a high degree of rationality and control (36) and does not focus heavily on how other people in AGYW's lives—such as partners, family members, and healthcare workers—could influence AGYW engagement in this model. Future research testing this model should assess whether and how such individuals influence AGYW use or non-use of this model's target behaviors. Lastly, as with all qualitative studies, our findings may not generalize to other AGYW within and beyond Kenya, especially those with more limited access to education (50), or to those living in areas with fewer AGYW-focused PrEP delivery initiatives.

## Conclusion

This formative research suggests that PrEP-experienced and PrEP-naïve AGYW in Kenya may be willing to engage in a peer-delivered HIVST and referral model, which could potentially be layered onto existing PrEP delivery programs. Whereas, HIVST has, to date, largely been used as a screening tool to identify individuals with HIV-positive status, this model primarily uses HIVST as a tool for motivating individuals, most of whom will test negative, to change their HIV risk behavior (e.g., increase condom use) and/or uptake PrEP (51, 52). The current proposed model refers AGYW to clinics, as that is where PrEP is primarily available in Kenya; however, to mitigate common access-to-care barriers faced by AGYW, implementers of this referral model may consider incorporating additional implementation strategies that could support follow-through on referrals, such as transportation vouchers (53), peer accompaniment to clinics (54), and linkages to PrEP programs, like DREAMS, that initiate and continue clients on PrEP in community-based safe spaces, outside of health facilities (55). Lastly, in addition to pilot testing this model and evaluating its acceptability, feasibility, and effect on HIVST and PrEP uptake, future research should explore variants of this model that may further reduce barriers to PrEP for AGYW, such as having peer providers distribute an initial supply of PrEP along with the HIVST kit (56) or refer peer clients to retail pharmacies that offer PrEP (57–59).

## Data availability statement

The qualitative data and descriptive statistics used in this study are not publicly available but are available upon reasonable request.

## Ethics statement

The studies involving human participants were reviewed and approved by Institutional Review Board of the University of Washington 116 (STUDY00009127); Scientific Ethics Review Unit (SERU) of KEMRI (0164/4004). The participants provided their written informed consent to participate in this study.

## Author contributions

Qualitative data collection was performed by EC. The codebook was designed, the analysis was conducted, and the first draft of the manuscript was written by MM and SDR. The analysis was reviewed and agreed on via census by MM, SDR, and KFO. All authors contributed to the design and development of the study. All co-authors provided feedback on the manuscript draft. All co-authors read and approved of the manuscript.

## Funding

Open access funding was enabled and organized by the Deutsche Forschungsgemeinschaft within the funding program Open Access Publikationskosten as well as by Heidelberg University. This study was funded by the United States National Institute of Mental Health (R00-MH121166, PI: KFO). KFO and KN additionally received financial support from the National Institute for Mental Health (R34-MH120106, PI: KFO; R01-MH113572, MPI: KN, N Mugo). The funders had no role in the design of the study, data collection and analysis, decision to publish, or preparation of the manuscript.

## Conflict of interest

The authors declare that the research was conducted in the absence of any commercial or financial relationships that could be construed as a potential conflict of interest.

## Publisher's note

All claims expressed in this article are solely those of the authors and do not necessarily represent those of their affiliated organizations, or those of the publisher, the editors and the reviewers. Any product that may be evaluated in this article, or claim that may be made by its manufacturer, is not guaranteed or endorsed by the publisher.

## Abbreviations

AGYW: adolescent girls and young women
ART: antiretroviral therapy
DREAMS, Determined, Resilient, Empowered, AIDS-free: Mentored and Safe
FSW: female sex worker
HIVST: HIV self-testing
IBM: Integrated Behavior Model
IDI: in-depth interview
IQR: interquartile range
KES: Kenya Shillings
MSM: men who have sex with men
PEP: post-exposure prophylaxis
PHRD: Partners in Health &
Research Development
PrEP: pre-exposure prophylaxis
RAST: Rapid Screening Assessment Tool
SERU: Scientific Ethics Review Unit
SSA: sub-Saharan Africa
USD: United States Dollars.
