# Supplementary material for: Assessing young Kenyan women's willingness to engage in a peer-delivered HIV self-testing and referral model for PrEP initiation: A qualitative formative research study
Source: Front Public Health. 2022 Oct 6;10:932948. doi: 10.3389/fpubh.2022.932948 (PMC9583529; doi:10.3389/fpubh.2022.932948)
Supplement: Supplementary file 1 [file Data_Sheet_1.docx]

# *Appendix 1: In-depth interview guides (PrEP-Experienced & PrEP-Naïve AGYW)*

| **1.0. Demographics** | **2.0. PrEP Rapid Assessment Screening Tool (RAST)** |
| --- | --- |
| I’d like to start by asking you some basic questions about yourself.   \| 1. How old are you? \| _________ years old \| \| --- \| --- \| \| 2. How many years of education have you completed? \| _________ years \| \| 3. What is your current occupation? \| _________________________ \| \| 4. About how much money do you make per month? \| _______ KSH per month \| \| 5. Thinking about everyone you live with, about how much money per month does your household make as a whole? \| _______ KSH per month \| \| 6. What is your current marital status? \| *Married*  *In a relationship, not married*  *Single, never married*  *Single, divorced or widowed* \| \| 7. Do you have any living children? \| *Yes*  *No* \| \| 7a. [If yes] How many living children do you have? \| *__________* children \| \| 8. Do you have close girlfriends whom you trust and confine in? \| *Yes*  *No* \| \| 9. [PrEP-Experienced] What year and month did you first start taking PrEP? \| *__ __ __ __ - __ ___*  *(Y Y Y Y) (M M)* \| \| 10.[PrEP-Experienced] Have you start and stopped PrEP since then? \| *Yes*  *No* \| | Now I would like to ask you some questions about your HIV status/risk:   \| 1. When did you last test for HIV? \| _________ months  *Never tested* \| \| --- \| --- \| \| 2. What is your HIV status \| *Negative*  *Positive*  *Unknown*  *Unwilling to disclose* \| \| 3. How many sexual partners have you had in the past month? \| __________ partners ______________________ \| \| 4. Do you have a primary sexual partner? \| *Yes*  *No* \| \| 4a. **[If yes]** What is his HIV status? \| *Negative*  *Positive*  *Unknown* \| \| ***In the past 6 months…*** \|  \| \| 5. Have you had sex without a condom with a partner(s) of unknown or positive HIV status? \| *Yes*  *No* \| \| 6. Have you engaged in sex in exchange for money or other favors? \| *Yes*  *No* \| \| 7. Have you been diagnosed with or treated for an STI? \| *Yes*  *No* \| \| 8. Have you used post exposure prophylaxis (PEP) two times or more? \| *Yes*  *No* \| |

| **3.0 In-depth Interview Guide**  *AGYW’s Willingness to Engage in a Peer-Delivered HIVST & Peer Referral to PrEP Intervention* | | |
| --- | --- | --- |
| *First, I would like to hear your thoughts about your community’s attitudes towards HIV and PrEP, as well as your relationship with your peers* | | |
| **Q1: Describe to me different attitudes towards HIV in your community.**   - *[Prompts]:* Describe to me attitudes toward HIV among your family members, among your peers? - How do these attitudes differ than those of individuals in your larger community? | | |
| **Q2: [PrEP-Experienced] Prior to starting PrEP, what different things had you heard about PrEP?**   - *[Prompts]:* How much knowledge or awareness of PrEP is there in your community? - What stories you have heard about PrEP (in community/with friends/at school, etc.)? - When people in your community talk about PrEP, how does the topic of PrEP come up? - What did they actually say? Can you cast your mind back to an instance when you heard somebody mention PrEP, and remember the conversation? - Sometimes people don’t communicate only with words, but also with their bodies or facial expressions. What was the emotion expressed? | **Q2: [PrEP-naïve] Pre-exposure prophylaxis or PrEP is a pill you can take every day that is nearly 100% effective at HIV prevention when used correctly. What (if anything) have you heard of PrEP?**   - *[Prompts]:* How much knowledge or awareness of PrEP is there in your community? - What stories have you heard about PrEP (in community/with friends/at school, etc.)? - When people in your community talk about PrEP, how does the topic of PrEP come up? - What did they actually say? Can you cast your mind back to an instance when you heard somebody mention PrEP, and remember the conversation? - Sometimes people don’t communicate only with words, but also with their bodies or facial expressions. What was the emotion express? | |
| **Q3: [PrEP-experienced] After hearing things PrEP, what (if anything) worried you in particular?**   - *[Prompts]:* Did you have any concerns about PrEP? Tell me more about these. - ***[If “no concerns”]*** If you heard negative things about PrEP, how did you process these stories or make sense of them? - Before starting PrEP, what were your thoughts on the side effects of PrEP? - What unanswered questions did you have about PrEP? | | **Q3:** **[PrEP-naïve] [*If you have heard of PrEP*] After hearing things PrEP, what (if anything) worried you in particular?**   - *[Prompts]:* Did you have any concerns about PrEP? Tell me more about these. - ***[If “no concerns”]*** If you heard negative things about PrEP, how did you process these stories or make sense of them? - What are your thoughts on the side effects of PrEP? - What unanswered questions do you have about PrEP? |
| **Q4: Describe to me your relationship with your peers.**   - *[Prompts]:* How much time do you typically spend with your peers each day? Week? - How large is this peer group? How many individuals do you feel particularly close with? - What types of things do you discuss with your peers? Do you discuss sex, family planning, HIV prevention methods? - Do you feel influenced by your peers and/or do you feel you have influence over your peers? Please describe. - Within your peer group, is there a clear leader(s) (e.g., an individual whose opinions usually matter more than others or whom people refer to for decision making)? If yes, who is this individual? Why are they the leader? - Do you think any of your close friends might be at risk of HIV infection? How many and why? - Would you feel comfortable talking to your peers about PrEP and HIV testing? | | |
| *Now I would like understand what things you do (including PrEP and others) to prevent yourself from HIV infection.* | | |
| **Q5: There are many different things that people at HIV risk can do to prevent infection, could you please describe some of these to me.**   - *[Prompts]:* Can you think of any other HIV prevention methods you might not have mentioned? - Which of these HIV prevention interventions that you have described do you or have you engaged in? - Have you stopped any of these interventions? Why? - Are there any things that your friends/peers do to prevent HIV infection that you don’t do? **[If yes]** What are these and why do you choose not to participate? - Are there any things you do to prevent HIV infections that your peers and friends don’t do? **[If yes]** Why do you think they choose not to participate? | | |
| **Q6: [PrEP-experienced] If you had to describe PrEP to a peer, how would you describe PrEP?**   - *[Prompts]:* What types of questions do you think your peers might ask you about PrEP? - Would you feel comfortable talking with your peers about PrEP? Why (not)? - What things might make it easier for you to talk with your peers about PrEP? | | **Q6: [PrEP-naïve] How would you feel if a peer approached you and asked you if you would like to start PrEP?**   - *[Prompts]:* What types of questions would you have for this peer about PrEP? - What concerns might you have about a peer approaching you to talk about PrEP? - How comfortable would you feel talking with your peer about PrEP? Why (not)? - What things might make it easier for you to talk with your peer about PrEP? |
| **Q7: Have you heard of HIV self-testing? [If no, describe HIVST] Can you share your thoughts or experiences with HIV self-testing?**   - **[If yes]** Where do you go to access HIV self-testing? Why? - *[Prompts]:* In what locations would you feel comfortable HIV self-testing? Why? - Would you prefer to HIV self-test alone or with others? Why? - Are you confident you could correctly take the test and interpret the test results? Why? - What support, if any, would you like with HIV self-testing? - Would you know where to go for follow-up care for HIV self-testing? If yes, where would you go and why? - [PrEP-experienced] If trained, would you feel comfortable sharing HIV self-testing with peers? Assisting them with HIV self-testing? | | |
| *Now I would like to hear about how you access PrEP care and your experiences with this care.* | | |
| **Q8: [PrEP-Experienced] Where do you access your PrEP care?**   - *[Prompts]:* What do you think about the clinics or medical settings where PrEP is offered? (e.g., staff, cleanliness, wait times, location, privacy, etc.) - Why do you choose to access PrEP at the location you do? (e.g., convenience, privacy, etc.)? - Where would you most prefer to access PrEP care? Why? - How often do you access PrEP care? What prompts you to access PrEP care? | | **Q8: [PrEP-naïve] If you were to start PrEP, where would you be most interested in accessing PrEP care?**   - *[Prompts]:* Why would you choose to access PrEP at that location? (e.g., convenience, privacy, etc.) |
| **Q9: Testing for HIV can be an emotional experience, what things (if any) do you do to help you regularly test for HIV?**   - *[Prompts]:* Do you find support from certain individuals? Do you seek a particular location or individual to help you HIV test? Please describe. - [PrEP-experienced] How might you support a peer if they wanted you to help them HIV self-test? Would you feel comfortable in this role? | | |
| *Now I would like to hear about your regular health behaviors (regarding HIV testing and PrEP) and the things you do to help maintain these behaviors.* | | |
| **Q10: [PrEP-Experienced] Taking a pill every day is hard, what strategies have you developed to help you consistently use PrEP?**   - *[Prompts]:* Do you use aids, such as your phone or an alarm, to help you take PrEP? - Do other individuals support you in your PrEP use? If so, who and how? - How would you feel disclosing your PrEP use to peers? - How would you feel about supporting close peers in their daily PrEP use? What might be challenging? | | **Q10: [PrEP-naïve] Taking a pill every day (as would be required for PrEP use) is hard, what strategies might you developed to help you consistently use PrEP?**   - *[Prompts]:* How might you feel if a close friend offered to help support you taking PrEP every day? What might be some of the pros and cons of this? |
| **Q11: Thinking into the future, for how many months or years do you think you might need to take PrEP?**   - *[Prompts]:* How did you come to this number? What things or life events might influence your future PrEP use? | | |
| *Miscellaneous* | | |
| **Q12: Is there anything else you would like to share with me today?** | | |
